# Analysis of Poly(thiourethane) Covalent Adaptable Network through Broadband Dielectric Spectroscopy

**DOI:** 10.1021/acsapm.2c01543

**Published:** 2023-01-06

**Authors:** B. Pascual-Jose, S. De la Flor, A. Serra, A. Ribes-Greus

**Affiliations:** † Institute of Technology of Materials (ITM), Universitat Politècnica de València (UPV), Camí de Vera, s/n, 46022València, Spain; ‡ Department of Mechanical Engineering, Universitat Rovira i Virgili (URV), Av. Països Catalans, 26, 43007Tarragona, Spain; § Department of Analytical and Organic Chemistry, Universitat Rovira i Virgili (URV), C/ Marcel·lí Domingo 1, 43007Tarragona, Spain

**Keywords:** covalent adaptable networks, broadband dielectric spectroscopy, dielectric spectra, poly(thiourethane), thermosets, charge transfer mechanism

## Abstract

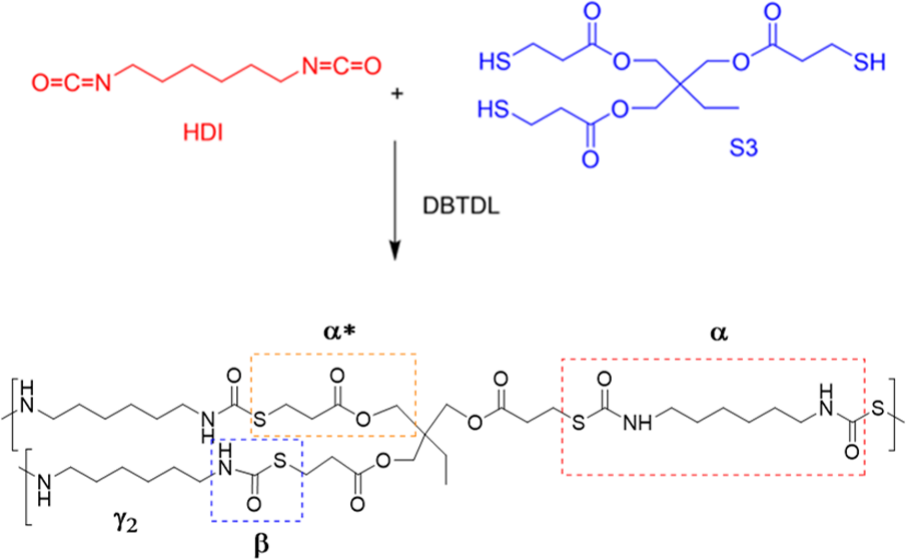

The dielectric spectra of the poly(thiourethane) network, HDI-S3, have been analyzed to know the nature and the cooperativity of each of the six dielectric processes observed. At low temperatures, γ_1_, γ_2_, and β dielectric relaxations were attributed to noncooperative local motions in the glassy state, in which apparent activation energies are 30, 36, and 60 kJ·mol^–1^, respectively. At higher temperatures, three dielectric relaxations are observed (α_Tg_, α*, ρ). The α_Tg_ relaxation is attributed to the glass transition, and it is overlapped with the α* relaxation. The molecular origin of α* relaxation is associated with the bond exchange reaction. Finally, the ρ relaxation is ascribed to the heterogeneity of the sample although its origin is uncertain. The DC conductivity (σ_DC_) is found to be an appropriate variable to analyze the bond exchange reaction. Accordingly, the HDI-S3 has a molecular exchange mechanism of dissociative nature.

## 1 Introduction

Thermosets are polymers with high dimensional and chemical stability over a broad range of temperatures. Nonetheless, their drawbacks are the uneasiness of being reshaped or reprocessed, which makes them very difficult to recycle. Covalent adaptable networks (CANs) are networked polymers that reduce the gap with thermoplastics by including reversible chemical bonds in the 3D structure. This type of chemistry allows for materials that display good mechanical properties at work temperatures, as thermosets do. Still, at the same time, they display good self-healing, weldability, and recyclability capacities, which thermosets do not possess.^1,2^

The viscoelastic behavior of CANs is determined by two temperatures, the glass transition (*T*_g_) and the bond exchange reaction, also called topological freezing temperature (*T*_v_). The former describes the long-range segmental motions. The latter is unique for CANs and distinguishes them from thermosets with permanent chemical bonds. *T*_v_ signals the onset of the transition from a viscoelastic solid to a viscoelastic liquid, and subsequently, the network can rearrange its topology. This occurs because, after *T*_v_, the timescale of the bond exchange reaction becomes shorter than the timescale of the material deformation.^3,4^ Furthermore, CANs can be classified according to the type of bond exchange mechanism. Accordingly, associative CANs are the ones where the bonds are broken but continuously formed again, whereas, in dissociative CANs, bond breakage dominates over bond formation.

In a first approximation, it could be thought that polythiourethanes (PTUs) may be compared to polyurethanes (PUs). However, the former displays several advantages than make them more desirable. For instance, properties such as biocompatibility, flexibility, excellent optical transparency, or a more homogeneous structure favor the appearance of relaxation processes in a narrower temperature range. Moreover, PTUs are formed through click-type reactions from isocyanates and thiols, and contrary to what occurs in polyurethanes, thiol-isocyanate reactions do not generate byproducts. It has been determined that a trans-thiocarbamoylation process is the origin of the networks’ vitrimeric-like behavior in PTUs, which, thanks to the presence of sulfur, could favor self-welding and stress dissipation. Therefore, a covalent adaptable network with a fast exchange mechanism is sought in synthesizing PTU containing dibutyltin dilaurate (DBTDL) as the catalyst.^5−7^

Broadband dielectric spectroscopy (BDS) is an established technique to study the dynamics of polymers, considering the response to an electrical perturbation field over a wide range of frequencies and temperatures, which provides information on large supramolecular systems and molecular motions. BDS is a valuable technique for the analysis of CANs because it can provide helpful insights into the nature of the bond exchange mechanism. Only a few examples are found in the literature exploiting this technique to go beyond the insights into these materials.^8−13^

Therefore, this work contributes to the characterization of CANs by analyzing the dielectric and conductive properties of a PTU vitrimer-like network through BDS. First, the molecular dynamics are fully analyzed to characterize all the dielectric processes paying special attention to the molecular motions that originate the trans-thiocarbamoylation reaction. Indeed, given the difficulties of directly measuring the topological freezing temperature and considering the high concentration of dipoles moving when the reaction occurs, BDS characterization should be able to provide an accurate view of the temperature range where the molecular motions associated with this chemical reaction are active. Second, the electric conductivity is analyzed to determine the associative or dissociative nature of the bond exchange mechanism since a direct correlation can be established between viscosity and DC conductivity. Additionally, the characterization of the PTU network is completed by studying the chemical structure through Fourier transform infrared spectroscopy (FTIR), and the assessment of the thermal properties is carried out through differential scanning calorimetry (DSC), thermogravimetric analysis (TGA), and dynamic mechanical analysis (DMA).

## 2 Experimental Procedure and Calculations

### 2.1 Materials and Preparation

**Scheme 1:**
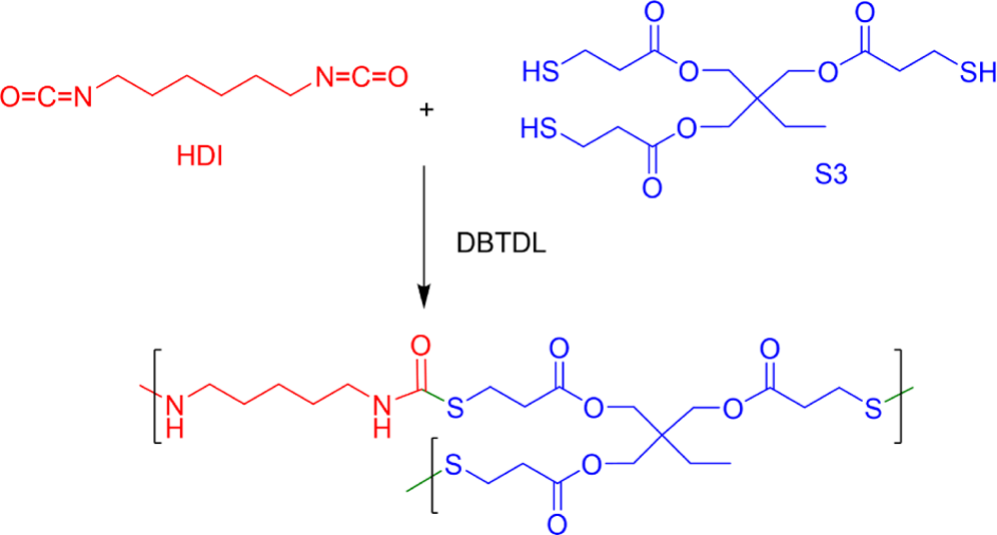
Synthesis of the HDI-S3 Network

Hexamethylene diisocyanate (HDI), trimethylolpropane tris(3-mercaptopropionate) (S3), and dibutyltin dilaurate (DBTDL) from Merck were used as received. Briefly, the sample (HDI-S3) was prepared, as shown in Scheme 1, by mixing stoichiometric amounts of HDI and S3 and adding a 4% w/w DBTDL as the catalyst, homogenized, and then cured in the oven at 333, 353, 373, and 423 K for 2 h at each temperature.^5^ The sample was dried for 3 h at 373 K before the measurement. The details of the preparation of the sample were explained elsewhere.^6^

### 2.2 Fourier Transformed Infrared Spectroscopy (FTIR)

The chemical structure was assessed through Fourier transform infrared spectroscopy (FTIR). Analyses were carried out in a Thermo Nicolet 5700 infrared spectrometer with an attenuated total reflectance (ATR) accessory. The spectra were collected from 4000 to 400 cm^–1^ at a resolution of 4 cm^–1^ along 64 scans. The spectra of three different locations of the sample were averaged. Backgrounds were collected, and results were processed through the Omnic Software.

### 2.3 Differential Scanning Calorimetry (DSC)

The differential scanning calorimetry (DSC) analyses were evaluated using Mettler Toledo DSC822e equipment. Aluminum capsules were filled with the samples, between 2 and 4 mg, and sealed. Then, they were subjected to a heating/cooling program with a rate of 5 K·min^–1^ over the 263 to 403 K temperature range under an inert atmosphere with a flow rate of 50 mL·min^–1^ of nitrogen.

### 2.4 Thermogravimetric Analysis (TGA)

The thermogravimetric analysis (TGA) was carried out with a Mettler Toledo TGA/STDA 851e setup. Samples with a mass between 2 and 5 mg were placed into 70 μL alumina capsules. An empty capsule was used as a blank to take the reference baseline. The analyses were performed with a heating rate of 30 K·min^–1^ over the 303 to 1073 K temperature range using an oxidative atmosphere with a flux of 50 mL·min^–1^ of oxygen.

### 2.5 Dynamic Mechanical Analysis (DMA)

DMA tests were conducted in shear mode with a small clamping assembly of 10 mm in diameter through a DMA/SDTA861e Dynamic Mechanical Analyzer from Mettler-Toledo (OH). Experiments were carried out in temperature step/frequency sweep mode from 303 to 523 K with isothermal steps of 5 K, between 10^–2^ and 10^2^ Hz.

### 2.6 Broadband Dielectric Spectroscopy (BDS)

The impedance measurements were conducted using a Novocontrol Broadband Dielectric Impedance Spectrometer (BDIS), connected to a Novocontrol Alfa-A Frequency Response Analyzer. The measurements were run in the frequency range of 10^–1^–10^–7^ Hz, at the temperature range 123 to 523 K. All the measurements were performed under isothermal conditions by increasing in steps of 10 K in the temperature range from 123 to 200 K and in steps of 2.5 K in the temperature range from 213 to 523 K. This change in the temperature step was made considering the narrow temperature range in which relaxation processes start in the HDI-S3 CAN. Thus, a better resolution of every dielectric process can be obtained. The sample electrode assembly (SEA) consisted of two stainless steel electrodes filled with the sample. Consequently, the resulting SEA was directly placed in the cell.

The dielectric spectra were analyzed in terms of the complex permittivity (ε*) using as many Havriliak–Negami (HN) functions as needed.^14−16^ All the characteristic parameters of each relaxation process were determined as shown in eq 1
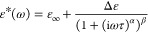
1where τ_HN_ is the Havriliak–Negami relaxation time, thus, the sub-index *k* represents the number of individual HN contributions. *a* and *b* are parameters corresponding to the width and asymmetry of the relaxation peak, respectively. Δε is the value of the relaxation strength.

The analysis of the temperature dependence of the relaxation times is performed in terms of an Arrhenius equation (eq 2) if the motion is noncooperative or through a Vogel–Fulcher–Tamman–Hesse (VFTH) equation (eqs 3 and 4) if the relaxation is of a cooperative origin.^17−21^
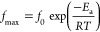
2
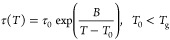
3
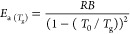
4where *f*_max_ refers to the maximum frequency, τ is the relaxation time, *f*_0_ and τ_0_ are pre-exponential terms, *B* is a constant, and *T*_0_ denotes the Vogel temperature. *T*_g_ is the glass transition temperature, *E*_a_ is the activation energy, and *R* is the ideal gas constant.

The response to an applied electric field of a polymer consists mainly of frequency-dependent and frequency-independent components. The former is ascribed to the DC conductivity and shows a frequency-independent plateau. In contrast, the latter is attributed to the AC conductivity and is characterized by a high dispersion at higher frequencies.^22^ This behavior can be modeled by Jonscher’s power law (eq 5).
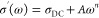
5where *A* is the pre-exponential factor, σ_DC_ is the frequency-independent value, and the *n*-parameter is a fractional exponent varying between 0 and 1.

## 3 Results

### 3.1 Chemical Structure

**Figure 1:**
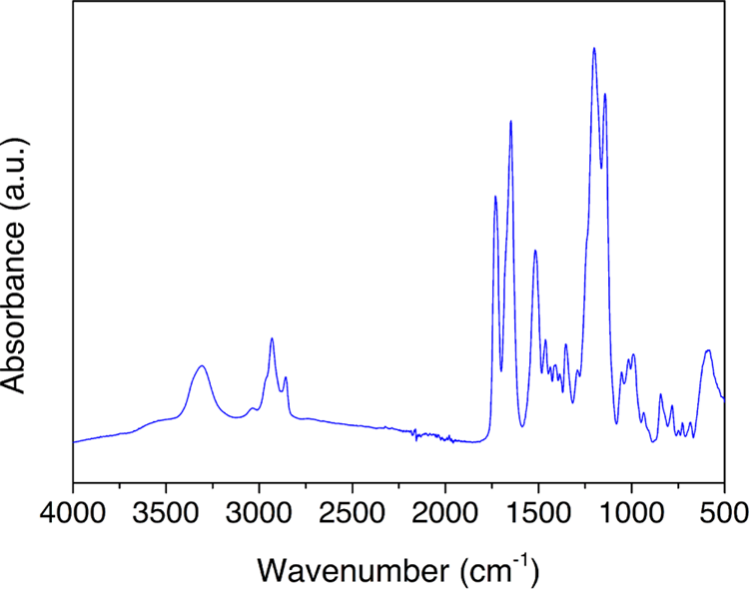
FTIR spectrum of the HDI-S3 sample.

The chemical structure of the HDI-S3 sample was assessed through the Fourier transform infrared spectroscopy (FTIRS), shown in Figure 1, to confirm that the PTU network’s formation is completed.

The C–H stretching band and the asymmetric and symmetric C–H stretching of CH_2_ were found at 3034 and 2932 cm^–1^, respectively. In addition, the C=O stretching corresponding to the ester linkage, present in the S3, was found at 1731 cm^–1^. Hydrogen bonded and non-hydrogen bonded C=O stretching bands of the thiourethane group were located at 1649 and 1680 cm^–1^, respectively. Moreover, N–H bending and N–H stretching bands, located at 1514 and 3303 cm^–1^, confirmed the thiourethane linkage. Besides, the wide bands between 3500 and 3000 cm^–1^ due to O–H stretching and the asymmetric peak around 1600 cm^–1^, corresponding to the bending band of water, acknowledged interactions with water molecules.^7^

### 3.2 Thermal Analysis

The thermal properties were characterized through differential scanning calorimetry (DSC). Accordingly, a controlled heating–cooling–heating program under an inert atmosphere was carried out. While the first heating scan erases the thermal history, the sample can be evaluated in the second heating scan, avoiding the specific effects of processing, storage, etc.

**Figure 2:**
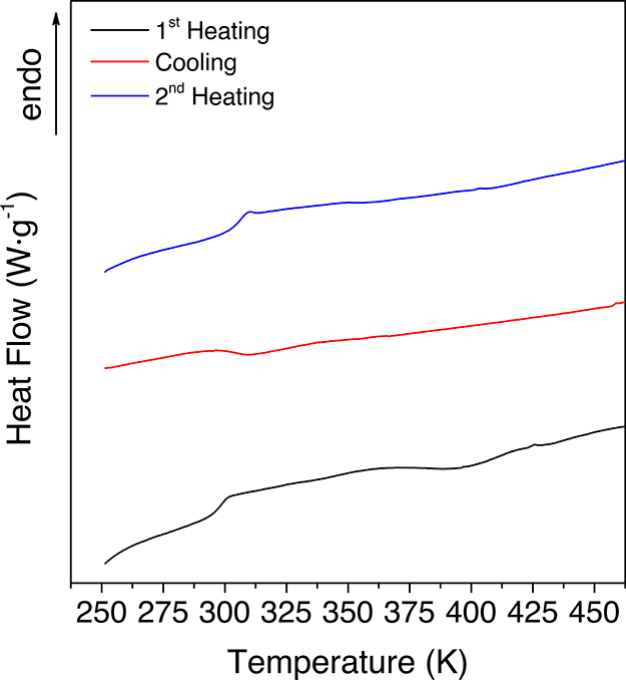
DSC thermograms of the first heating, cooling, and second heating for the HDI-S3 sample.

Nonetheless, in Figure 2, the first heating, second heating, and cooling curves are plotted. The glass transition, attributed to the flexible backbone of the HDI-S3, is found around 310 K. This value agrees well with other results for similar networked structures.^23^

**Figure 3:**
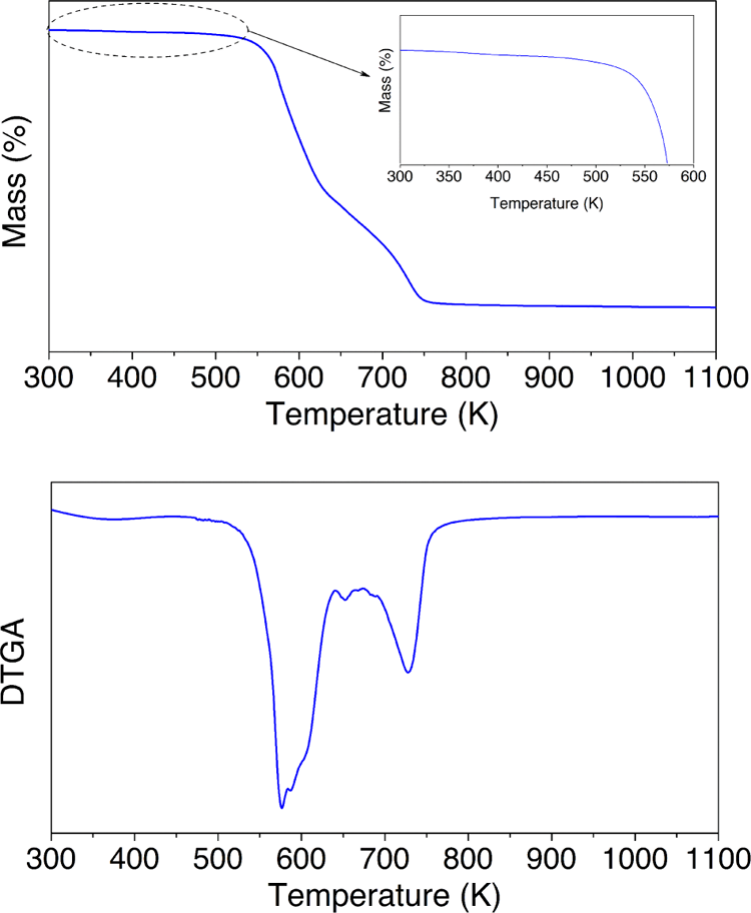
Thermogravimetric curves for the HDI-S3 sample.

The thermal stability of the sample was assessed through thermogravimetric analysis (TGA). The samples were subjected to a dynamic thermal program under an oxidative atmosphere. The weight loss as a function of time was studied through thermogravimetric thermograms and differential curves, as shown in Figure 3, where several mass-loss stages are found.

The first small-scale mass-loss stage may be due to the evaporation of remnant-bound humidity, but it is a few percentage. The subsequent more prominent mass-loss stage is found above 523 K and is associated with the elimination of carbonyl sulfide during the decomposition process of the thiourethane.^23,24^ Furthermore, the next stage related to the peak around 613 K is attributed to the β-elimination processes of the esters of the thiol structural units.^5,7^ At last, the mass loss between 673 and 773 K agrees with the degradation of the backbone.

**Figure 4:**
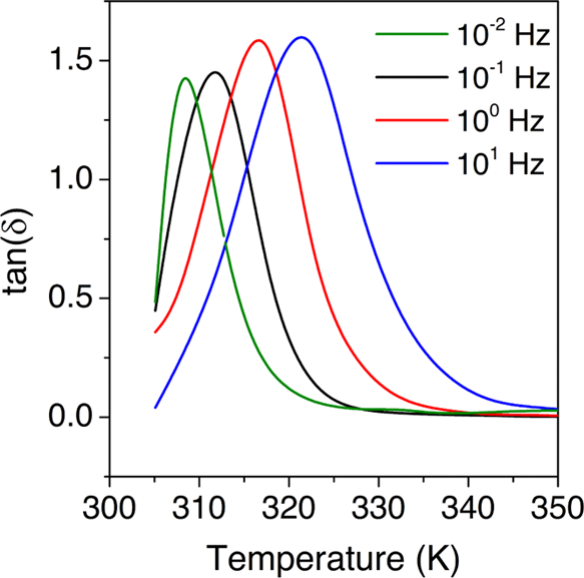
Loss tangent at several frequencies for the HDI-S3 sample.

The thermomechanical properties were assessed through DMA, obtaining the tan δ evolution with temperature, displayed in Figure 4. Regarding molecular mobility, it can be appreciated an increment in the intensity and width of the relaxation as the temperature increases. This means that there is an increment in the number of molecules in motion, which is characteristic of cooperative processes.

Therefore, this prominent peak, found in the tan δ spectrum at several frequencies, is ascribed to the glass transition. The peak temperature is found to vary between 308 and 322 K from 10^–2^ to 10^1^ Hz, respectively. Furthermore, an estimated apparent activation energy value of 216 kJ·mol^–1^ is obtained. This value is in line with the *T*_g_ obtained by DSC (310 K), very similar to the peak of the tan δ curve at low frequencies. Besides, it agrees with the *T*_g_ obtained in previous thermomechanical analyses of similar PTU networks.^6^

### 3.3 Analysis of the Dielectric Spectra

**Figure 5:**
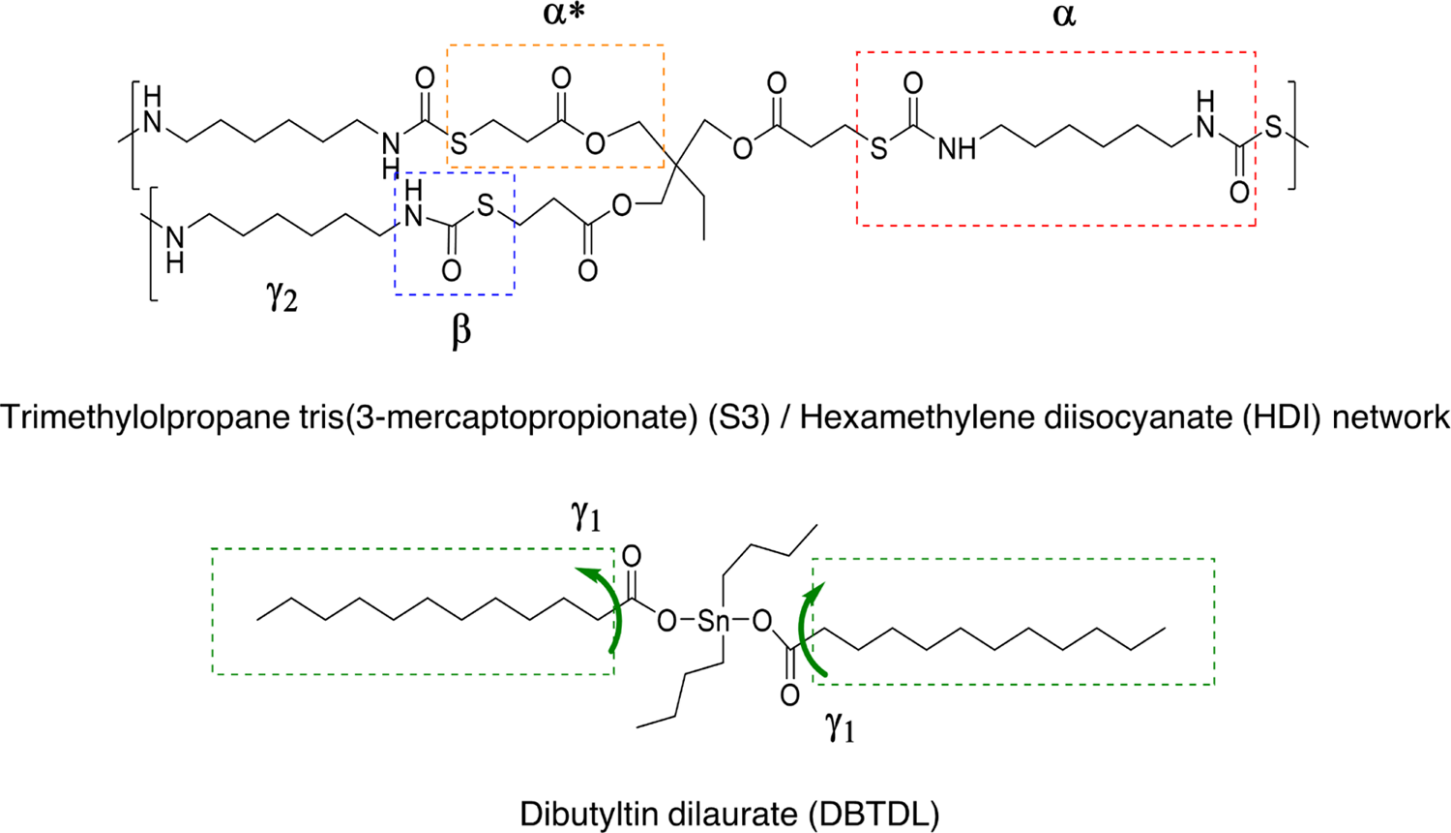
Molecular structure of the HDI-S3 and DBTDL. This figure also includes the proposed molecular origin of all the dielectric processes.

The analysis of the dielectric spectra is performed through the complex permittivity formalism (ε*). The frequency and temperature dependence of its real (ε′) and imaginary (ε″) parts are studied in the frequency range *f* = 10^–2^–10^7^ Hz from 123 to 523 K. The dielectric spectrum of HDI-S3 spectrum initially displays six relaxations. The first region is located at low temperatures and labeled in order of increasing temperature as γ_1_, γ_2_ and β, respectively. The other relaxations are located at mid-to-high temperatures, and the three dielectric relaxations have been labeled as α_Tg_, α*, and ρ, which may be related to the glass transition, bond exchange, and interfacial polarization, respectively. Figure 5 shows the molecular structure, and the highlighted groups may be the origin of these dielectric relaxations.

**Figure 6:**
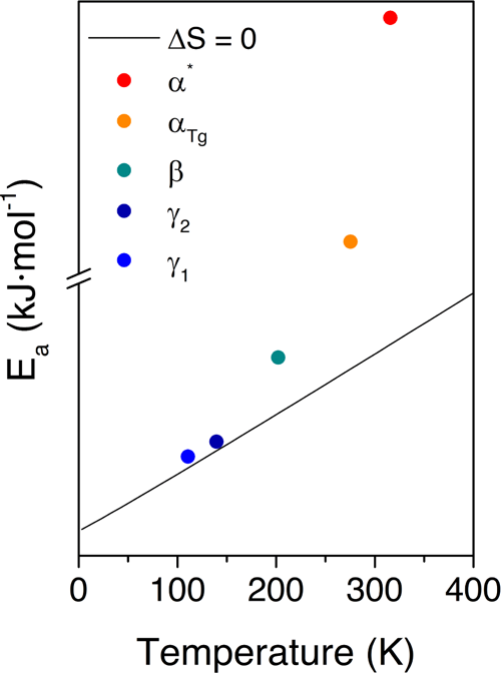
Eyring graph for all the dielectric relaxations of the HDI-S3 sample at a frequency of 1 Hz.

In Figure 6, the macromolecular origin of the dielectric relaxations is assessed through the Eyring model as derived by Starkweather.^25^ Accordingly, the *E*_a_ values close to the zero-entropy line, determined as *E*_a_ = *RT*[22.92 + ln *T*],^26^ can be considered of intramolecular (or noncooperative) origin since the entropy’s role can be disregarded for this type of molecular relaxation. On the contrary, values far from the zero-entropy line are classified as of intermolecular (or cooperative) origin because their departure from the zero-entropy values indicates that the contribution of the entropy is significant. Therefore, it cannot be disregarded. Consequently, the dielectric relaxations occurring in the low-temperature region (γ_1_, γ_2_) are the ones with a noncooperative origin since their values are close to the zero-entropy line.

Note that the β relaxation, despite being a low-temperature process, has its *E*_a_ value far from the zero-entropy line. This is because a certain degree of cooperativity might be involved in this molecular motion, probably due to its proximity to the glass transition, as already seen in other polymers such as polyesters or polyethylene.^27−29^

On the other hand, the dielectric relaxations located in the high-temperature region (α_Tg_ and α*) are the cooperative molecular motions because their *E*_a_ values lie very far from the zero-entropy ones.

To further assess the observed dielectric relaxations, the dependence of the relaxation times with respect to the temperature must be analyzed. Consequently, the spectrum has been divided into two different relaxation zones corresponding to the different macromolecular nature of the motions.

### 3.4 Thermal Dependence and Macromolecular Origin of the Low-Temperature Relaxation Zone

**Figure 7:**
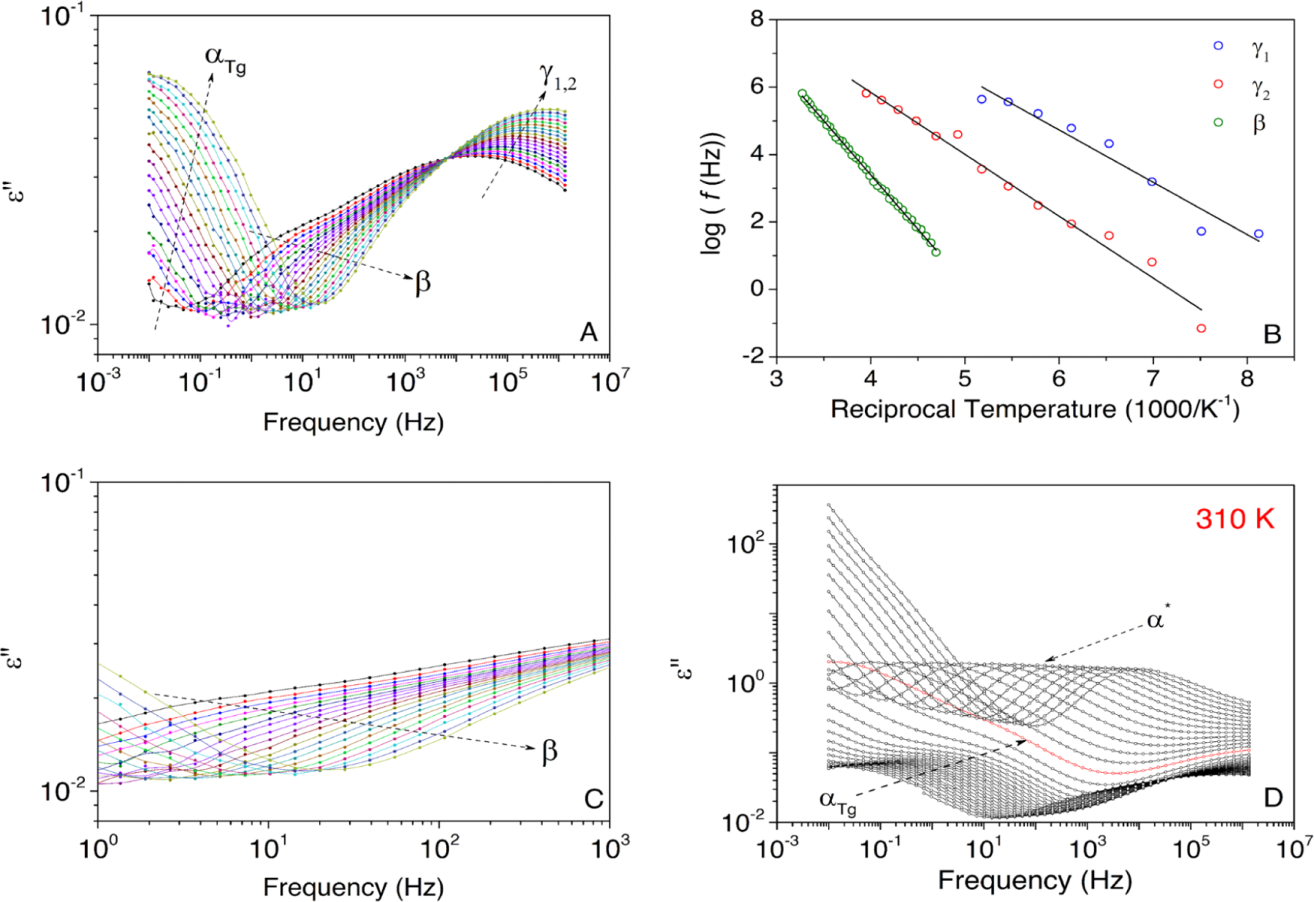
(A) Isothermal curves of the imaginary part of the complex permittivity (ε″) for the HDI-S3 sample in the temperature range from (top) 133 to 203 K; (B) Arrhenius map for the low-temperature relaxation zone of the HDI-S3. Solid lines represent the fitted lines; (C) detailed view of the isothermal curves of the imaginary part of the complex permittivity (ε″) for the HDI-S3 sample in the temperature range from 213 to 233 K; (D) isothermal curves for the HDI-S3 sample in the temperature range between 255 and 343 K. The red line signals the onset temperature for the bond exchange reaction.

The low-temperature region consists of three dielectric relaxations labeled as γ_1_, γ_2_, and β, in order of increasing temperature. Figure 7A–C shows isothermal curves in the temperature range from 153 to 253 K. It is observed that at very low temperatures, the γ_1_ and γ_2_ processes are mostly indistinguishable since they overlap each other. Subsequently, they must arise from a very similar molecular motion, and a very similar apparent activation energy should be expected. The β-process arises at higher temperatures. This dielectric process displays a lower intensity and is visible in a narrow temperature range because it is quickly overlapped by another dielectric process appearing at low frequencies.

Figure 7B plots the relationship between the relaxation time and the temperature for the three dielectric processes occurring at low temperatures. All of them have a noncooperative behavior (γ_1_, γ_2_, and β). Subsequently, the thermal dependence has been adequately assessed using an Arrhenius function, and the corresponding parameters for the best fit are gathered in Table 1.

**Table 1:** Activation Energy (*E*_a_) of the Low-Temperature Dielectric Relaxations of the HDI-S3 Sample

relaxation	intercept	*E*_a_(kJ·mol^–1^)	*R*^2^
γ_1_	14.05 ± 0.92	30 ± 1	0.945
γ_2_	13.18 ± 0.33	36 ± 1	0.986
β	16.26 ± 0.08	62 ± 1	0.996

Regarding its molecular origin, these relaxations have also been observed in the dielectric spectra of hyperbranched polyurethanes, and given the temperature range where it occurs, some authors ascribed them to local motions of particular functional groups.^30^ Several works have analyzed the functional group motions that give rise to these relaxations in different polymers, but it is an issue highly controversial. Some authors consider that the hydroxyl groups are the origin of the motion of the γ_1_ and γ_2_ relaxation in polyesters.^29,31^ More recently, it has been proposed that its molecular origin might be linked to the ether oxygen-containing segments.^32^ However, in this polymer (HDI-S3), these functional groups are not present; consequently, they are not the origin of these relaxations. Other authors assign these dielectric relaxations to local motions of (CH_2_)*_n_* sequences, which they are included in the HDI-S3 structure.^27,28,33,34^

It is not possible to ascribe the molecular origin giving rise to a dielectric process without considering in the first place the molecular structure of HDI-S3, which is displayed in Figure 5. Thus, the γ_1_ and γ_2_ may be related to the motion of the (CH_2_)*_n_*, which could be found at the catalyst DBTDL pivoting thanks to the carbonyl group and at the end groups of the cross-linked structure, respectively. Both relaxations have a similar apparent activation energy. The obtained values are 30 and 36 kJ·mol^–1^, respectively. These values are in line with the values found by other researchers that range from 30 to 43 kJ·mol^–1^.^32,34,35^

Concerning the β relaxation, there are also some discordances regarding its molecular origin. For instance, Yu et al. suggested that this process can be attributed to a local motion of oxygen-containing ether groups.^36^ Castagna et al. proposed that this molecular motion results from the reorientational motions of water molecules.^37^ Other researchers suggest that the β relaxation originated from the motion of the polar carbonyl groups with attached water molecules.^38,39^ Nonetheless, the notion that this dielectric process is severely affected by humidity is of general consensus.^36−39^

Indeed, despite samples being dried before the measurements, residual water is still left due to strong interactions between water molecules and the polar thiourethane and ester groups,^34,36,39^ as confirmed by the thermogravimetric curves shown above. Consequently, the only zones that can be hydrophilic are the carbonyl groups located in the thiourethane and ester moieties, as described in Figure 5. Since this would take place in the carbonyl groups, this relaxation should not display a significant intensity, and this apparent activation energy is higher than the γ_1_ and γ_2_ relaxations. This hypothesis is validated by the isotherms displayed in Figure 7A,B.

### 3.5 Thermal Dependence and Macromolecular Origin of the High-Temperature Relaxation Zone

**Figure 8:**
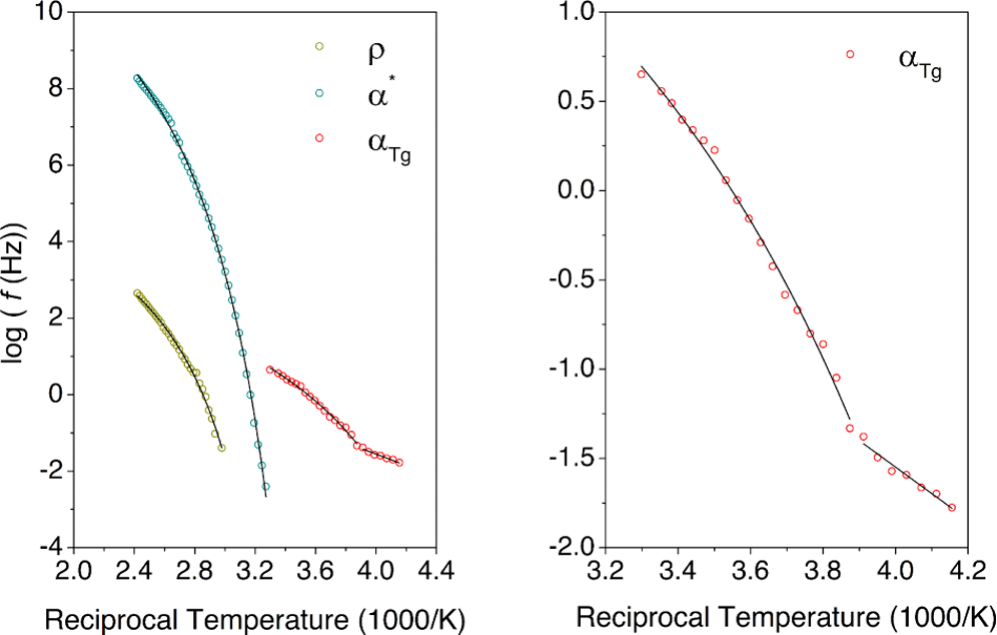
Arrhenius plot of the high-temperature relaxation zone for the HDI-S3 sample (left) and detailed view of the α_Tg_ (right). Solid lines represent the fitted lines.

The high-temperature region displays three dielectric processes (α_Tg_, α*, and ρ), as shown in Figure 8. As aforementioned, at high temperatures, the dielectric spectra of this PTU are formed by the relaxation labeled α_Tg_, which may be related to the glass transition and the ρ-process that could be ascribed to the interfacial polarization (Maxwell–Wagner–Sillars (MWS)) due to its heterogeneous microstructure.^32,36−43^ Both processes are overlapped by a prominent dielectric process, labeled as α*, which may be associated with the bond exchange reaction.

As shown in Figure 8, the relationship between the relaxation time of each one of these relaxations and the temperature is not linear, and consequently, these dielectric processes have been fitted through a VFTH model. The corresponding results are gathered in Table 2.

**Table 2:** Best Fit of the HN Parameters for the HDI-S3

temperature (K)	*a*_HN_	*b*_HN_	Δε
265.65	0.43	0.98	0.38
270.65	0.43	1	0.39
285.65	0.46	1	0.39
295.65	0.44	1	0.51
298.15	0.44	1	0.55

Figure 7D shows the isothermal curves between 243 and 333 K with the low-intensity signal related to the α_Tg_ process. Additionally, the α_Tg_ process is overlapped by the dielectric process associated with the bond exchange reaction, as indicated by the red line. This result was already expected because the HDI-S3 is completely cured,^6^ which means the cross-linking process is completed. Subsequently, large segmental motions are not expected, while the bond exchange reaction supposes a much greater motion of dipoles.

**Figure 9:**
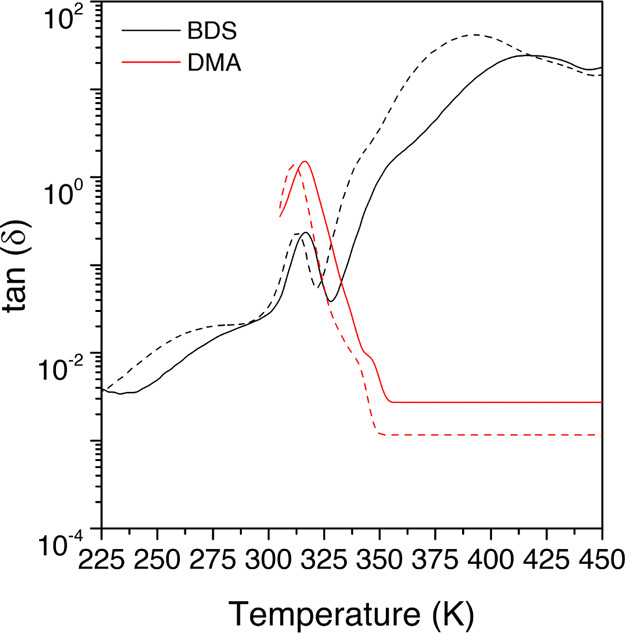
Comparison between the loss tangents measured with DMA (red line) and BDS (black line) at 10^–1^ Hz (dashed lines) and 10^1^ Hz (full line), respectively.

In Figure 9, the comparison between the isochrones at two frequencies with the equivalent dynamic mechanical data is presented, showing a significant agreement between them. Therefore, this corroborates the ascription of this molecular motion to the glass transition.

The Havriliak–Negami shape and strength parameters (*a*_HN_, *b*_HN_, and Δε) corresponding to the α_Tg_ are gathered in Table 2. A general review of these parameters indicates that the values of the strength parameter increased with increasing temperature. At low temperatures, this increment is slow, whereas at high temperatures, it increases significantly. This is expected due to the interaction with the α* process. In addition, the shape parameters indicated that the relaxation distribution of the α_Tg_ process was similar to the Cole–Cole function. This type of behavior has already been observed in other thermoset polymers, for instance, the work by Núñez-Regueira et al. in a cured epoxy diglycidyl ether of bisphenol A (DGEBA).^44^

Figure 7D also shows the more prominent α*, which may be attributed to the molecular dynamics of the bond exchange reaction occurring in this temperature range.^45,46^ Furthermore, another feature that allows identifying α* is the fact, as shown in Figure 7D, that the dielectric process decreases with the temperature.

From previous results on the viscoelastic properties of HDI-S3, it has been found that *T*_v_ occurs at temperatures higher than the glass transition.^6^ Through dynamic mechanical analysis, the *T*_v_ temperature was estimated to be 369 K.^6^

Generally, it is accepted that it is very difficult for bond exchange reactions to take place below the *T*_v_.^1−3,47−49^ Recently, the consensus has broken down since more experimental techniques are used in the study of CANs, and subsequently, new insights arise. For example, Hubbard et al. discuss that the bond exchange reaction can occur at very low temperatures. However, the *T*_v_ acts as the onset temperature where the timescales of the process become significant.^48^ Additionally, Schoustra et al. reported that actual *T*_v_ values could be much lower than the initially estimated value.^50^ This proves that the molecular dynamics associated with the bond exchange reaction can occur at temperatures lower than those previously estimated. Consequently, when analyzing the data displayed in Figures 7D and 9, one can conclude that the molecular dynamics associated with *T*_v_ initiate approximately at 310 K. Above this temperature, the timescale of the bond exchange reaction becomes more relevant than the segmental motions, which originate the glass transition and become a significant process.

As previously mentioned, the relationship between the relaxation time of α* relaxation and the temperature is not linear. As seen in Figure 9, thus, this dielectric process has been fitted through a VFTH model. Table 3 displays the best fit for the VFTH model for the cooperative motions. The value obtained for the fragility parameter (*D*) denotes a fragile behavior. This is expected provided the nature of the dielectric process. The value for the free-volume coefficient of the α* process presents an expected normal value, considering that for most systems, this value lies in the interval 0.025 ± 0.005.^51^

**Table 3:** VFTH Parameters and Derived Parameters for the α_Tg_, α*, and ρ Dielectric Processes

process	log *f*_0_	*T*_VFTH_ (K)	*D*	*R*^2^	Φ_Tg_	*E*_a_(kJ·mol^–1^)
α_Tg_	2.38 ± 0.31	257.66 ± 8.33	0.54 ± 0.25	0.997	0.04	268^a^
α*	11.81 ± 0.08	269.33 ± 0.58	4.70 ± 0.10	0.999	0.03	540^a^
ρ	5.65 ± 0.24	275.92 ± 4.43	3.51 ± 0.42	0.995	0.06	273^a^

a The *E*_a_ value of each process is calculated using *T*_g_, *T*_v_, and *T*_ρ_, respectively.

On the contrary, the ρ process displays a higher value (0.06), which can be considered that is overlapped with the bond exchange mechanism, which is already active. Furthermore, *T*_VFTH_ agrees with the value found in Figure 9 that has been used to estimate the temperature range for the molecular dynamics related to *T*_v_. Regarding the apparent activation energy (*E*_a_), the value for the α* process is 513 kJ·mol^–1^. This value accounts for the energy associated with the complete molecular dynamics originated in the network once *T*_v_ is reached and can be obtained, thanks to the ability of the BDS technique to provide raw data in several orders of magnitude. Other works reported the apparent activation energy necessary to initiate the process, assessed by mechanical or dielectric analysis using the Arrhenius equation. It is very important to note that the apparent activation energy calculated using the VFTH equation depends on the temperature (eq 4), unlike the apparent activation energy calculated using the Arrhenius equation (eq 2), which is independent of it, and therefore, both values are not comparable. However, the analysis of the dielectric spectrum does not allow determining whether the process associated with the bond exchange reaction is dissociative or associative. To discern the nature of this process, many works asses the decrease in viscosity through dynamic mechanical analysis (DMA).^2,52−58^ In the dielectric analysis, its equivalent property may be the conductivity (σ).^59−62^ Thus, a deep study of the electric conductivity was carried out.

### 3.6 Analysis of the Electric Conductivity

**Figure 10:**
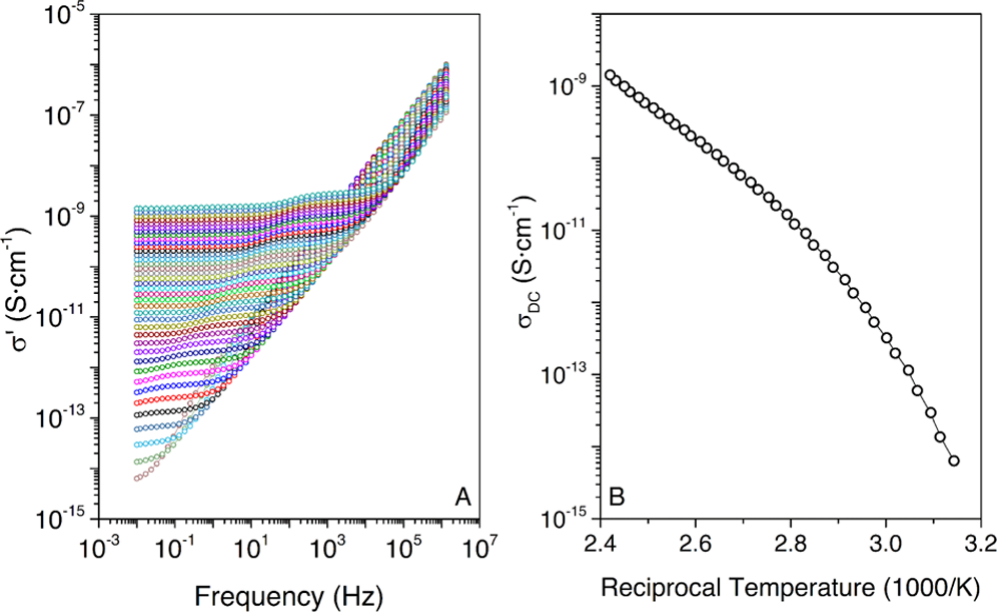
(A) Real part of the complex conductivity (σ′) of the HDI-S3 material from 318 to 413 K; (B) σ_DC_ curve for the HDI-S3 material.

The isothermal curves for the real part of the complex conductivity (σ*) are plotted in Figure 10A from 318 to 413 K. The Jonscher’s model (eq 5) is used to determine the DC conductivity (σ_DC_). The values corresponding to the best fit for a selected range of temperatures are gathered in Table 4.

**Table 4:** Jonscher’s Parameters for Several Temperatures

temperature (K)	σ_DC_	*A* × 10^15^	*n*	*R*^2^
361	2.20 × 10^–11^	8.84 × 10^1^	1	0.904
366	3.67 × 10^–11^	5.61 × 10^3^	1	0.998
378	1.12 × 10^–10^	8.31 × 10^2^	1	0.997
403	6.96 × 10^–10^	8.65 × 10^1^	1	0.977
413	1.42 × 10^–09^	6.51 × 10^1^	1	0.959

The *n*-parameter displays a value of 1, which means that the long-range pathways necessary for ion transfer are not altered. The values of σ_DC_ augment with increasing temperature, which signifies a thermally activated process. Moreover, in Figure 10B, the temperature range from 318 to 413 K is observed. The σ_DC_ values are influenced by the cooperative dielectric relaxations that may be coupled with the ion transferring. Therefore, it shows the influence on the electrical conductivity of the α* and ρ relaxations. Furthermore, an estimation of the apparent activation energy is done and a value of 129 kJ·mol^–1^ is obtained. In a previous article, an assessment of the apparent activation energy of the process was performed through stress relaxation. However, in the literature,^9,12^ the apparent activation energy obtained through BDS for stoichiometric thiol systems was found to be 110–120 kJ·mol^–1^.

To determine the nature of the dynamic bond exchange, in many works, the temperature dependence of the viscosity is determined through dynamic mechanical analysis (DMA).^2,52−58^ Accordingly, an associative behavior is assigned if a linear (Arrhenius-like behavior) relationship is observed. On the contrary, if the relationship is nonlinear (VFTH-like behavior), the bond exchange mechanism is estimated to be dissociative.

Concerning BDS, although there are several methodologies to assess the behavior of the bond exchange reaction,^10,12,63,64^ no general consensus has been reached yet. For instance, Luo et al.,^47^ due to the lack of a theory based on the dielectric relaxation’s energy landscape with consistent physical principles, proposed a model based on the existing differences between the apparent activation energy and the actual activation energy of the bond. In this model, the populations of dissociated and associated states are defined by the energy difference between them (*E*_bind_ = *E*_diss_ – *E*_asso_). Accordingly, dissociative CANs are predicted to display binding energy values lower than (2–4)*RT*, while associative CANs will display values around 4*RT*. Although low values of the binding energy do not necessarily mean a dissociative bond exchange mechanism, the model proved capable to explain, at least in the fundamental physical aspect, why some CANs, which are supposed to have a dissociate bond exchange mechanism, display an associative behavior instead. In Podgórski et al.,^9^ a different approach is taken. It is argued that an associative exchange mechanism when active would not promote network depolymerization, and thus, the frequency response of the electric modulus (M*) would be constant. Therefore, although there is still more research to be done to develop new methodologies that fully describe the nature of the bond exchange reaction, BDS capability to provide information in a wide range of temperatures and frequencies might be fundamental in the physical characterization of CANs.

In thermosets, the curing process of epoxy resins is monitored through the ionic conductivity.^59−62^ Several works have demonstrated the validity of this approach. For instance, Simpson et al. demonstrated that in thermosets, ionic conductivity could be estimated to be inversely proportional to the viscosity in the hydrodynamic regime.^65^ Furthermore, Friedrich et al. argued that the conductivity (σ_DC_) is directly proportional to the ion mobility during the curing process when no variations in the ionic concentration are found. Nonetheless, the charge carrier density varies during the reaction and it limits the suitability of the general principle stating that the ionic conductivity is directly linked to the medium viscosity.^66^

Krouse et al. measured the two components of the conductivity, namely, the ion mobility and the number of mobile charge carriers, in an epoxy/amine formulation before and during the curing process over a similar range of viscosity through ion time-of-flight and dielectric measurements. The results showed that ion mobility is the parameter that best correlates with the viscosity, both with changes in temperature and network growth.^67^ Therefore, the σ_DC_, displayed in Figure 10B, can be considered to analyze whether the molecular exchange mechanism in a covalent adaptable network is of dissociative or associative origin when BDS is used.

Accordingly, the obtained results suggest that the HDI-S3 polymer has a dissociative molecular exchange mechanism.^2,45,52−58^ This is in line with previously reported results regarding the HDI-S3 material. By heating this sample in a polar solvent like dimethyl sulfoxide (DMSO), the sample was dissolved, indicating that bonds were broken.^5,6^ However, the dissociation of thiourethane groups to isocyanates and thiols is completely reversible and very fast, which finally leads to a vitrimer-like behavior.

## 4 Conclusions

The dielectric spectra of the poly(thiourethane) network, HDI-S3, have been analyzed. Six dielectric processes have been found, three of them at low temperatures (γ_1_, γ_2_, and β), and the rest are located at higher temperatures (α_Tg_, α*, ρ). The low-temperature processes are attributed to a diverse range of local motions in the glassy state. Moreover, the data provided by DMA and DSC allowed us to properly determine the glass transition temperature and, therefore, to identify the α_Tg_ dielectric process associated with the glass transition. Furthermore, the α* relaxation is attributed to the molecular dynamics that arises from the bond exchange reaction. Accordingly, a temperature range has been found for this exchange reaction, from 303 to 435 K, but the onset temperature where the timescale of the bond exchange becomes more relevant is located at 310 K. Finally, the ρ process is attributed to interfacial polarization due to the heterogeneity of the sample.

Through the analysis of the temperature dependence of the DC conductivity (σ_DC_), the present article would suggest that the HDI-S3 polymer has a dissociative molecular exchange mechanism. Traditionally, the majority of studies performed using DMA only covered a range of relaxation times of 3–4 orders. In the present study, however, a wider range of relaxation times, up to 10 orders, have been used. Consequently, a comprehensive characterization of molecular mobility and electric conductivity through broadband dielectric spectroscopy provides useful information for the physical characterization of CANs.
